# The impact of stigma on health care-seeking behavior in military personnel with mental health challenges

**DOI:** 10.1097/MS9.0000000000004569

**Published:** 2025-12-16

**Authors:** Sana Rasheed, Eisha Kashif, Rida Arif, Amna Shakeel, Rubaisha Saleem, Ahmed Asad Raza, Abedin Samadi

**Affiliations:** a Department of Medicine, Jinnah Sindh Medical University, Karachi, Pakistan; b Department of Medicine, Dow University of Health Sciences, Karachi, Pakistan; c Department of Medicine, Kabul University of Medical Sciences Abu Ali Sina, Kabul, Afghanistan

**Keywords:** help-seeking behavior, military mental health, PTSD, stigma, veterans

## Abstract

Prolonged armed conflict has raised significant concern about the mental health of military personnel and veterans. Conditions such as post-traumatic stress disorder and depression affect approximately 14–16% of U.S. service members deployed to Afghanistan and Iraq. Other prevalent mental health issues include suicide, traumatic brain injury, substance use disorder, and aggression. Despite the availability of mental health services, stigma remains a major barrier to care, with nearly 60% of affected personnel avoiding treatment. This review examines how stigma influences help-seeking behavior in military populations. A systematic search of PubMed and Scopus was conducted using terms such as “stigma,” “health care-seeking behavior,” “military personnel,” and “mental health,” limited to English-language studies published between January 2000 and November 2024. Additional data were obtained from cited references and recent conference materials. The findings indicate that stigma in the military manifests in three main forms: public, self-, and institutional stigma, each of which contributes uniquely to reluctance toward seeking care. There is a consistent negative correlation between stigma and treatment seeking. Programs emphasizing education, peer support, and leadership engagement have shown promise in improving attitudes. Initiatives such as the Real Warriors Campaign and evidence-based treatments like Trauma-Informed Guilt Reduction contribute to reducing stigma and promoting recovery. A multifaceted approach involving policy reform, anti-stigma training, and culturally competent interventions is essential to improving mental health outcomes and service engagement among military members.

## Introduction

Over two decades of prolonged armed conflict highlighted significant public and professional concerns about the mental health challenges faced by veterans and service members. Among the highly recognized conditions, post-traumatic stress disorder (PTSD) and depression are well known, which affect approximately 14–16% of U.S. service members deployed to Afghanistan and Iraq^[1,2]^. Although the mental health challenges in this population go beyond PTSD and depression, including suicide, traumatic brain injury, substance use disorder (SUD), and aggression^[3]^. These issues often arise from battlefield encounters, extended deployments, separation from family, and the built-in pressure of military life. The impact of military service on mental health is significant, as deployments and combat exposure increase the risk of developing mental health disorders. Additionally, extended separation from family, regular reassignment, and the stress of adapting the military culture add to the psychological strain^[4]^. While combat and deployment have high association with mental health conditions, challenges can also arise during routine armed forces duty. Mental health symptoms do not always present immediately, but certain periods, such as near battlefield situations or shifts from military to non-military life, are especially demanding for service members and their families^[5]^.

**HIGHLIGHTS**

- Stigma prevents 60% of military from seeking mental health care.
- Public, self, and institutional stigma blocks help-seeking behavior.
- Untreated stigma-linked illness increases suicide and substance risk.
- Education, peer support, and telehealth reduce stigma barriers.
- Campaigns and therapies improve care and reduce stigma in military.

Mental health is an essential aspect of overall well-being, yet many individuals, particularly in the general population, do not pursue care despite access to proven treatments. A significant number of people with mental health challenges lack necessary assistance, mainly because of lack of awareness and the stigma associated with mental health disorders^[6,7]^.

Stigma surrounding mental health remains a major obstacle in pursuing help and accessing professional treatment. Many people with mental health problems hesitate to get treatment due to the anxiety of being judged, misunderstood, or discriminated against. This stigma often stems from public misunderstanding and negative stereotypes about mental illness, which can create emotional distress and social withdrawal for those affected^[8,9]^. About 60% of military personnel who experience mental health problems refrain from seeking help, yet many of them could benefit from professional treatment. Across military studies, one of the most commonly cited obstacles to help seeking for mental health problems is concerns about stigma^[10]^. This shows that stigma weighing heavy over the lives of veterans battling mental health issues. Despite their bravery in service, many face severe criticism and unfair treatment because of their struggles. This stigma acts as a barrier, preventing them from seeking the vital support they need^[10]^. This narrative review examines how stigma affects mental health care-seeking behavior among military personnel and evaluates strategies to reduce these barriers. It addresses the following key questions:

1. How do different forms of stigma influence willingness to seek care?
2. What aspects of military culture, organizational norms, and leadership practices contribute to mitigating stigma?
3. How do confidentiality concerns and institutional policies shape trust in mental health services?
4. Which evidence-based interventions, including education, leadership engagement, and policy reforms, effectively reduce stigma and improve care-seeking?

By exploring these questions, this review aims to consolidate current evidence and guide targeted approaches to support mental health help seeking within the military population. Additionally, the review will evaluate the effectiveness of mental health awareness campaigns in encouraging military personnel and veterans to attain appropriate care.

## Methodology

### Search strategy

A comprehensive search was conducted in PubMed and Scopus to identify studies examining the influence of stigma on mental health care-seeking among military personnel. The search covered the period from January 2000 to November 2024 and used combinations of the terms “stigma,” “mental health,” “military personnel,” “veterans,” and “help-seeking behavior.” To ensure thorough coverage of the literature, reference lists of included studies and related reviews were manually screened for additional relevant publications. All steps of the search and reporting process adhered to the Transparency In The Reporting of Artificial Intelligence in Research (TITAN-AI) 2025 guidelines to ensure responsible use of AI tools in the conceptualization, drafting, and refinement of the review^[11]^.

### Inclusion and exclusion criteria

Eligible studies were those involving active-duty service members, veterans, reservists, or other military-affiliated personnel and assessing any form of stigma – public, self, or institutional – as it related to mental health help-seeking, treatment utilization, or perceived barriers to accessing psychological care. Studies presenting empirical findings in quantitative, qualitative, or mixed-methods formats were included, provided they were published in English within the defined timeframe. Studies were excluded if they focused solely on civilian populations, explored stigma unrelated to mental health, lacked sufficient empirical data to inform the review aims, consisted only of conference abstracts without full-text availability, or were non-research commentaries or opinion papers. These criteria ensured that only studies directly contributing to understanding stigma-related barriers in military mental health care were retained.

### Study selection process

**Figure 1.**
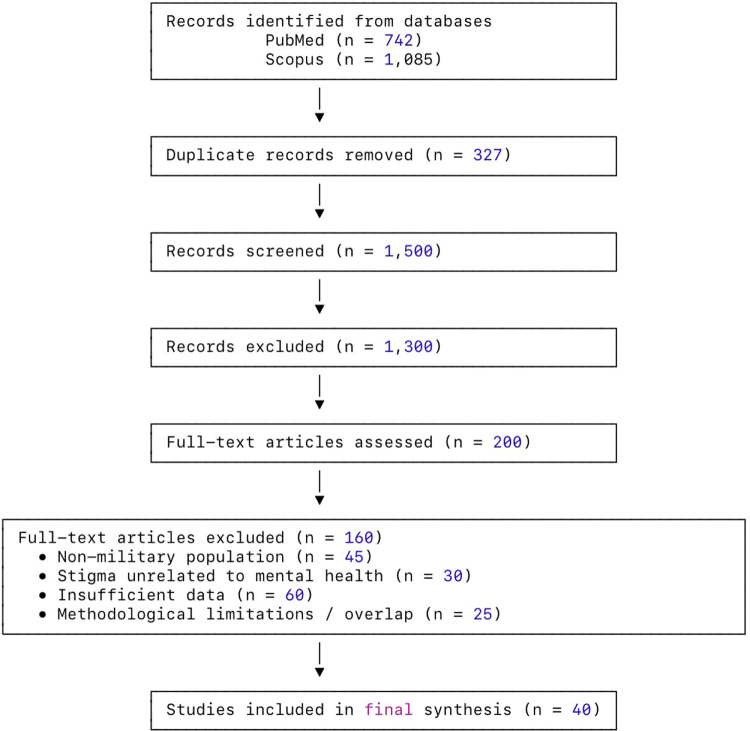
PRISMA flowchart.

The initial search yielded 1827 records (742 from PubMed and 1085 from Scopus). After removing 327 duplicates, 1500 unique records remained and were screened by title and abstract. Of these, 1300 were excluded for not meeting the eligibility criteria. Full-text assessment was performed on 200 articles, resulting in the exclusion of 160 studies due to reasons such as the use of civilian populations, exploration of unrelated stigma constructs, insufficient data on mental health help seeking, or methodological limitations including unclear design or overlapping data sources. Following this multistage process, 40 studies met all criteria and were included in the final synthesis. The structured approach enhances clarity and meets the reviewer’s request for improved transparency regarding article selection. The detailed study selection process is illustrated in the PRISMA flow diagram (Fig. 1).

### Quality assessment and bias considerations

A concise quality and bias assessment was performed to strengthen the rigor of the review. Because the included studies varied in design, a broad, narrative appraisal was used rather than formal tools such as NOS or ROBINS-I. Each study was examined for clarity of aims, appropriateness of methodology, adequacy of sample selection, transparency of data collection, and coherence between results and conclusions. Potential biases – including selection bias, self-report bias, recall bias, inconsistent measurement of stigma, and limitations inherent to cross-sectional designs – were identified and considered when interpreting findings. Heterogeneity in stigma definitions and assessment instruments was also acknowledged as a source of conceptual and methodological bias that may influence comparability across studies.

## Main Text

### Prevalence of mental health challenges in military personnel

Military personnel, including both active-duty and veteran soldiers, experience a high prevalence of mental health challenges attributed to the distinct stressors inherent in military service. Although PTSD is the most commonly observed disorder in this population, other issues such as depression, anxiety, and substance abuse are also comorbidities seen alongside PTSD^[12,13]^. A meta-analysis conducted by Hines *et al* in 2014 showed that 7.1% of all US, Canadian, and British soldiers who were in service in Afghanistan suffered from PTSD^[14]^. Furthermore, a longitudinal cohort study of entry-level US military personnel found that the prevalence of diagnosed depression increased from 11.4% in the year preceding deployment to 15% within 12 months following return from Iraq or Afghanistan, based on systemic medical record review^[15]^. SUDs, particularly alcohol misuse, also continue to be a concern with higher risks in veterans who are male, unmarried, and younger than 25 years of age^[16]^. A systematic narrative review examined the prevalence of psychiatric disorders among the West African military, highlighting a high burden of SUDs^[17]^. Alcohol use disorder demonstrated a particularly high prevalence, affecting approximately 76% of individuals^[18]^. The prevalence of cannabis use disorder was reported at 5.8%^[19]^, while non-medical use of prescription opioids disorder was observed in 3.6% of personnel^[20]^.

Various risk factors contribute to the frequency of mental health disorders in military personnel. The most significant predictors of post-deployment PTSD are accredited to the frequency and intensity of combat exposure. A United Kingdom study highlighted several additional risk factors, including lower rank, being unmarried, low education, facing low morale, and lack of unit social support^[21]^. The risk is further heightened in the absence of post-deployment psychological and social support^[22]^. Separation from families, repeated relocations, extended lengths of deployment, and combat stress – along with exposure to life-threatening situations – increase the risk of depression in both active-duty and veteran military personnel^[4]^. Experiencing injury in combat or witnessing death is strongly associated with suicide risk within the first 6–12 months after leaving the military particularly among veterans^[23]^. Moreover, the change from structured military life to civilian life for veterans presents its own difficulties, with many struggling to reintegrate into society and find employment.

**Figure 2.**
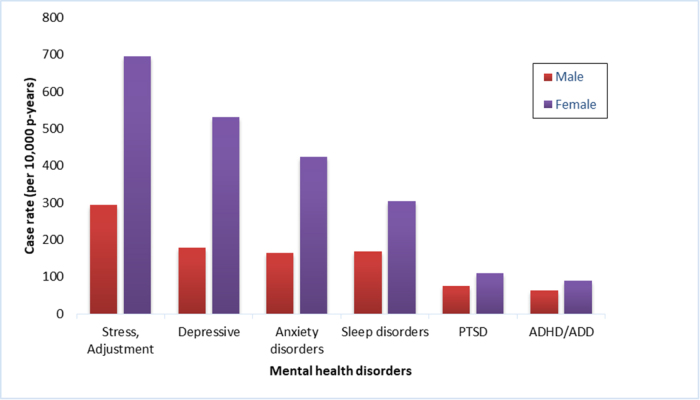
Case rates of the most common mental health disorders by sex, deployed soldiers, U.S. Army, 2008–2013.

A study published in 2022 highlighted the prevalence of mental health illnesses among US soldiers deployed to combat zones during 2008–2013. Figure 2 summarizes the results^[24]^.

### Stigma and its types

Stigma is frequently characterized as negative beliefs and attitudes toward people experiencing mental health challenges, often stereotyping them as incompetent and weak, leading to them being discriminated against and treated unfairly^[25]^. There is evident contrast between the mental health care systems of the military and civilian communities, and even though stigma is persistent in both, military values like strength and resilience that have been ingrained in a soldier’s mind make it challenging for them to seek treatment^[26]^. Stigma is further grouped as public stigma, self-stigma, and institutional stigma, each playing a distinctive role in the obstacles faced by people suffering from mental health challenges^[9]^. Prejudiced societal perceptions result in public stigma, also known as perceived stigma, and the unjust labeling of people with psychological illnesses as incompetent, which contributes to their feelings of exclusion or ostracization by society^[9,27]^.

Self-stigma is when individuals internalize the public’s biased opinions, giving rise to shame, embarrassment, and reduced self-esteem^[9,25,27]^. The subsequent feeling of inferiority discourages the person from seeking assistance for their mental health issues^[25]^. Armed force members may adopt the military cultural labels incorporating them in their minds as a personal weakness to seek mental health treatment, further exacerbating the internalized stigma and the subconscious fear of being avoided or rejected by important people in their personal lives^[26]^.

However, personal beliefs are not the only factor in perpetuating stigma; institutional structures also play a significant role. Institutional stigma is when the company’s organizational structure is not designed to accommodate an individual who wants to receive mental health care^[28]^. The system places disadvantages for people that take the initiative to receive care in the form of career ramifications and labeling soldiers as unfit for service, demonstrating a lack of support from the institution itself^[29]^. In an effort to evade the stereotype that seeking professional care often brings, military personnel are frequently deterred from engaging in help-seeking behavior^[9]^.

### Impact of stigma on healthcare-seeking behavior in military personnel

A significant factor that may be delaying people from seeking help is the strong stigma associated with mental illness. It has a particular effect on the military community considering the emphasis placed on resilience and persistence in the face of mentally taxing circumstances.

Numerous studies show that among military personnel, stigma around mental health is negatively correlated with treatment-seeking behaviors and their utilization^[30]^. A cross-sectional study conducted among members of the Japan Ground Self-Defense Force utilized the 10-item Attitudes Toward Seeking Professional Psychological Help Scale–Short Form to assess attitudes toward help-seeking behavior. The study found that both public stigma and self-stigma were inversely associated with help seeking, indicating that higher levels of stigma were linked to a reduced likelihood of accessing mental health services^[31]^. Activities related to seeking help and patients’ compliance with mental health treatment have been associated with stigma regarding mental health^[32]^.

Military personnel with mental health issues often delay deploying to mental health care seeking with fear of being viewed as less engaging, less self-assured, and emotionally unstable. This makes them believe that others in the military will build a negative impression and stigmatize people who seek professional mental health help due to which preconception, prejudice, and disparity against those seeking professional psychological care can result. When neglected, this frequently results in a rise in the prevalence of serious mental health issues including anxiety, depression, sleep disorders, PTSD symptoms, etc^[33]^.

In contrast, in a study done by Rosen *et al*, it was found that stigma did not deter veterans with PTSD from approaching psychotherapy and paradoxically a higher grade of stigma even reported more rigorous involvement from those already involved in psychotherapy^[34]^. Furthermore, in another study, concerns about stigma were apparent but did not appear to sway treatment-seeking behavior. This finding, however, was limited by a lack of formal validation of the method used to evaluate stigma^[35]^.

According to recent research, military personnel who recognized a need for mental health support but did not seek treatment were approximately 3.7 times more likely to attempt suicide (adjusted odds ratio = 3.65) over the deployment-to-postdeployment period^[36]^. Control procedures during their military service, including disciplinary proceedings, health issues like discomfort and pain, leadership disputes, duty zone transfers, rank reductions, or administrative departure etc., are few challenges that members may encounter while serving in the military. All these variables may raise the incidence risk of suicide, yet treatment is frequently delayed because mental health care is stigmatized^[4]^.

Consequently, the stigma linked to military personnel seeking medical help – especially for mental health issues – can greatly affect their willingness to pursue necessary care. By addressing this stigma, the military can enhance the overall well-being and health of service members, benefiting both them and the military as a whole.

### Interventions to reduce stigma

Raising awareness through educational programs is not only critical in lowering stigma by encouraging help-seeking behavior, but it also plays an important role in preventing suicide among military personnel. To promote early intervention, programs like the US Air Force’s suicide prevention^[37]^ emphasize mental health education and encourage mental health literacy. A similar program is employed by the Canadian forces^[38]^, which includes a multi-faceted strategy to ensures that a service member’s career or future chances are not adversely affected and that timely care is sought.

Seeking help is a sign of strength, not weakness. One useful approach is obtaining firsthand accounts from military members who have received mental health treatment^[39]^. This helps humanize the problem and decreases anxiety about criticism that can affect one’s career. Additionally, workshops and online courses debunk myths and provide relevant support by identifying mental health symptoms early so all personnel can comprehend the need for early intervention.

Furthermore, a cardinal role in shaping attitudes toward military health care is played by leadership and peer support programs. These programs draw attention to psychological well-being and foster a culture of support. When commanding officers and peers openly discuss mental health, it reduces stigma and encourages help seeking. Real Warriors Campaign (Psychological Health Center of Excellence, n.d.) is one such program, which includes personal recovery stories from military personnel to break down stigma.

Another important factor that may hinder access to timely mental health care is confidentiality. Therefore, policies that allow telehealth consultations anonymously are required. Also, mental health professionals should be embedded in military units to help members seek care without any fear of repercussions.

Off-base counseling and protecting medical records are other confidential care options that further encourage early intervention. In post-deployment health assessments, self-report surveys are included, which sometimes fail to evaluate the full extent of psychological stress faced due to social desirability bias. Due to concerns of stigma, military members underreport symptoms. In contrast, anonymous surveys yield more accurate insights by minimizing the fear of judgment^[40]^.

Evidence-based therapies are also crucial in raising awareness and addressing mental health issues among military personnel. One such therapy is Trauma-Informed guilt reduction therapy, designed to reduce combat-related guilt and stress^[41]^. It showed promising results in reducing PTSD, depression, and post-traumatic guilt; however, further research is needed to confirm its efficacy due to limitations with small sample size and thus decreased generalizability.

## Critical analysis of study limitations

While the reviewed studies demonstrate a negative relationship between stigma and mental health, several methodological and contextual limitations across the literature influence the interpretation of this study. As a result, the direct association between the stigma and military help seeking cannot be definitively established. Although recent studies and correlations have been reported consistently, it remains unclear whether high stigma reduces help seeking or whether avoidance of mental health services reinforces the perception of stigma. Since most studies are derived from U.S. active-duty male personnel, with comparatively fewer studies involving women, ethnic and racial groups, or non-Western minorities, this restricts the findings’ application to larger international military contexts. It may mask stigma processes that are specific to a person’s gender or culture. Most of the studies examined stigma alone without taking into consideration organizational and environmental factors that may interact, such as leadership styles, unit cohesion, past disciplinary experiences, or the perception of the secrecy of mental health care. Thus, this might suggest that stigma has a stronger or weaker effect than it actually does, without considering social and economic factors. Collectively, these limitations present significant factors to take into account. Even if the data repeatedly indicate that stigma prevents people from obtaining mental health treatment, the methodological flaws show that additional thorough, representative, and long-term studies are required to confirm and improve these conclusions.

## Future directions

Significant research gaps remain, particularly in the area of gender-specific stigma, as evidenced by the lack of research on female veterans, limited inclusion and focus on racial and ethnic differences, and the underrepresentation of non-US populations in existing studies. These areas should be the focus of further research, alongside an urgent need for longitudinal studies to better understand how stigma and mental health outcomes evolve over time within diverse military populations.

A promising future direction involves leveraging emerging neurobiological insights to enhance stigma-reduction interventions. Stigma operates as a chronic social stressor, with measurable biological effects, including dysregulation of the hypothalamic-pituitary-adrenal axis observed in minority stress contexts, such as blunted cortisol responses to standardized psychosocial stress tests^[42]^. At the neural systems level, discrimination has been linked to altered connectivity in salience and threat regulation networks, particularly the amygdala-prefrontal circuitry, which plays a central role in stress appraisal and regulation^[43]^. Moreover, epigenetic changes such as methylation in stress-responsive genes like NR3C1 have been associated with social stress exposure and predict attenuated cortisol reactivity^[44,45]^. By integrating these neurobiological mechanisms into intervention design, stigma-reduction strategies for military populations can be more precisely targeted and potentially more effective.

## Conclusion

Countless soldiers and veterans refrain from seeking help due to the fears of being deemed weak or facing professional consequences. In order to prevent the exacerbation of prominent mental health conditions such as PTSD, depression, anxiety, and SUDs among the military population, efforts to reduce the stigma surrounding these concerns is essential. Addressing this issue therefore warrants a multifaceted approach aimed at enhancing mental health awareness and education among military personnel by implementing comprehensive policies including evidence-based anti-stigma media campaigns, structured educational workshops, and accessible online training programs. Additional strategies – such as ongoing support and training for military personnel and their families, peer support programs featuring testimonials from military personnel who have received mental health treatment, and anonymous telehealth consultations and surveys – can help foster a more supportive and informed military culture, ultimately improving the overall well-being of service members. Despite the strengths of this narrative review, several limitations must be recognized. First, considering the largely US-centered literature used in this study, the findings may not fully translate to military populations in other countries with different cultural, organizational, and healthcare structures and thus limit generalizability. Furthermore, the use of only published peer-reviewed studies also raises the risk of publication bias which may overestimate the impact of stigma and omit studies with neutral or opposing findings.

## Data Availability

No data is available.
